# Moderate and severe traumatic brain injury: abbreviated injury scale scoring and coding of traumatic axonal injury from early MRI

**DOI:** 10.1186/s12873-025-01319-4

**Published:** 2025-08-20

**Authors:** Ingrid Aune Bergstrøm, Oddvar Uleberg, Anne Vik, Anne-Mari Holte Flusund, Anne Katharina Köster, Sozaburo Hara, Marianne Dahlhaug, Kent Gøran Moen

**Affiliations:** 1 Department of Clinical and Molecular Medicine, Faculty of Medicine and Health Sciences, Norwegian University of Science and Technology (NTNU), Trondheim, N-7491 Norway; 2 Department of Emergency Medicine and Pre-Hospital Services, St. Olavs Hospital, Trondheim University Hospital, Trondheim, N-7006 Norway; 3 Department of Circulation and Medical Imaging, Faculty of Medicine and Health Sciences, Norwegian University of Science and Technology (NTNU), Trondheim, N-7491 Norway; 4 Department of Neuromedicine and Movement Science, Faculty of Medicine and Health Sciences, Norwegian University of Science and Technology (NTNU), Trondheim, N-7491 Norway; 5 Department of Neurosurgery, St. Olavs Hospital, Trondheim University Hospital, Trondheim, N-7006 Norway; 6 Department of Radiology, Møre and Romsdal Hospital Trust, Sjukehuset Nordmøre og Romsdal, Hjelset, N-6450 Norway; 7 Department of Orthopaedics, Oslo University Hospital, Oslo, N-0450 Norway; 8 Department of Radiology, Vestre Viken Hospital Trust, Drammen Hospital, Drammen, N-3004 Norway; 9 Department of Radiology and Nuclear Medicine, St. Olavs Hospital, Trondheim University Hospital, Trondheim, N-7006 Norway

**Keywords:** Multiple trauma, Craniocerebral trauma, Trauma severity indices, Injury severity score

## Abstract

**Background:**

In this large prospective study of patients with moderate and severe traumatic brain injury (TBI) examined with early magnetic resonance imaging (MRI), we aimed to explore the differences in the occurrence and degree of traumatic axonal injury (TAI) reported on early MRI compared with (1) *the original Abbreviated Injury Scale (AIS)-TAI coding* and (2) *a pragmatic AIS-TAI coding* approach. The latter approach also allowed the assignment of TAI codes to patients with short periods of coma, sedated patients and patients with concurrent mass lesions, which are exclusion criteria in the original AIS-TAI scoring.

**Methods:**

A total of 311 patients with moderate (n=156) or severe (n=155) TBI, aged 8–70 years, admitted to a regional Level 1 trauma center and examined with early head MRI within six weeks (median 9 days) were included. Consultants in radiology reported TAI and TAI grades using the MRI sequences fluid-attenuated inversion recovery, diffusion-weighted imaging and either T2* gradient echo or susceptibility-weighted imaging. The AIS head score was reported according to *the original AIS-TAI coding* and using *a pragmatic AIS-TAI coding* approach.

**Results:**

Of all patients, 80% (n=249) had TAI lesions on early MRI. Using *the original AIS-TAI criteria,* only four patients (1%) were eligible to receive a TAI code, and they all had TAI grade 1 on early MRI. Using *a pragmatic AIS-TAI coding* approach, 50% (125/249) of the patients with TAI on MRI received a TAI code as part of their AIS head score.

**Conclusion:**

Our study demonstrates a substantial discrepancy in the proportion of patients diagnosed with TAI as part of the AIS head score, especially when *the original AIS-TAI coding* criteria were used but also when the *pragmatic AIS-TAI coding* was used, compared with findings of TAI on early MRI. Hence, we suggest a revision of the TAI coding in the AIS Dictionary, which is more reliant on findings on early MRI, which is increasingly performed in patients with moderate–severe TBI.

**Clinical trial number:**

Not applicable.

## Background

Traumatic brain injury (TBI) is one of the most common causes of death and disability in young people [1]. Patients with TBI are classified as mild, moderate or severe on the basis of their level of consciousness at admission to the hospital [2], which is usually assessed with the Glasgow Coma Scale (GCS) score [3].

Traumatic axonal injury (TAI), also known as diffuse axonal injury (DAI), is an important type of injury in TBI [4]. Before the 1980s, TAI was considered a stretch injury due to a primary axotomy, and thus a histopathological diagnosis that was closely associated with severe outcomes, including death [5]. In the last decades research has shown that a primary axotomy is not what most often occur, but rather the stretch injury induces a biochemical cascade inside the axon, resulting in bulb formation. If the injury is severe enough, it can over hours to days result in a secondary axotomy, but if it is not so severe, the axon might also heal [4]. Since small capillaries also are found along the axons, the stretch injury can also cause leakage of erythrocytes into the parenchyma. The increasing technological development and use of magnetic resonance imaging (MRI) have provided increased sensitivity in detecting TAI, and the abovementioned bulb formation and leakage of erythrocytes into the brain parenchyma can now be detected on MRI of TBI patients of all severities, even mild TBI [6, 7]. Usually, TAI lesions cannot be detected with CT [6, 8], and hence MRI is the modality of choice. The first histopathological classification system for TAI was proposed by Adams et al. in 1989 [9] and later adapted for use on MRI by Gentry et al. [10]. This standard TAI grading based on Adams and Gentry has in the last decades been used for grading of TAI on clinical MRI; grade 1 (TAI in the white matter of the hemispheres or cerebellum), grade 2 (TAI in the corpus callosum) and grade 3 (TAI in the brainstem). For patients with moderate and severe TBI, early MRI is now considered an important supplement to CT in the early post-traumatic phase for better visualization of parenchymal brain damage and for prognostication [11, 12].

The Abbreviated Injury Scale (AIS) is an anatomical injury severity scoring system developed by the Association for Advancement of Automotive Medicine and is commonly used to grade injury severity in trauma patients [13]. In AIS, injuries in different body regions are given a 6-digit unique numerical identifier (based on anatomical location) and a severity score ranging from one (minimal injury) to six (maximal injury), where scores ≥ 3 are considered serious. The AIS score for the body region head (AIS head score) has been suggested used for classifying TBI [14], but studies have shown large differences in severity scoring for TBI in particular, especially between institutions [15, 16]. Bågenholm et al. highlighted that the non-compliance between TAI diagnosis in the AIS Dictionary compared to the radiological reports are leading to incorrect coding [15]. This is because TBI patients with coma lasting less than six hours, sedation or concurrent mass lesion, are ineligible to be assigned with TAI codes according to the AIS Dictionary. With the increasing use of early MRI in the evaluation of patients with moderate-severe TBI, there is reason to believe that the differences in AIS head coding practice will be even larger in the future.

Thus, the primary aim of this study was to assess the differences in the occurrence and grade of TAI from early MRI compared with how TAI should have been coded using the strict *(1) original AIS-TAI coding* and how TAI was scored in practice with a more *(2) pragmatic AIS-TAI coding* approach, developed and used in consensus at our institution.

## Methods

**Fig. 1:**
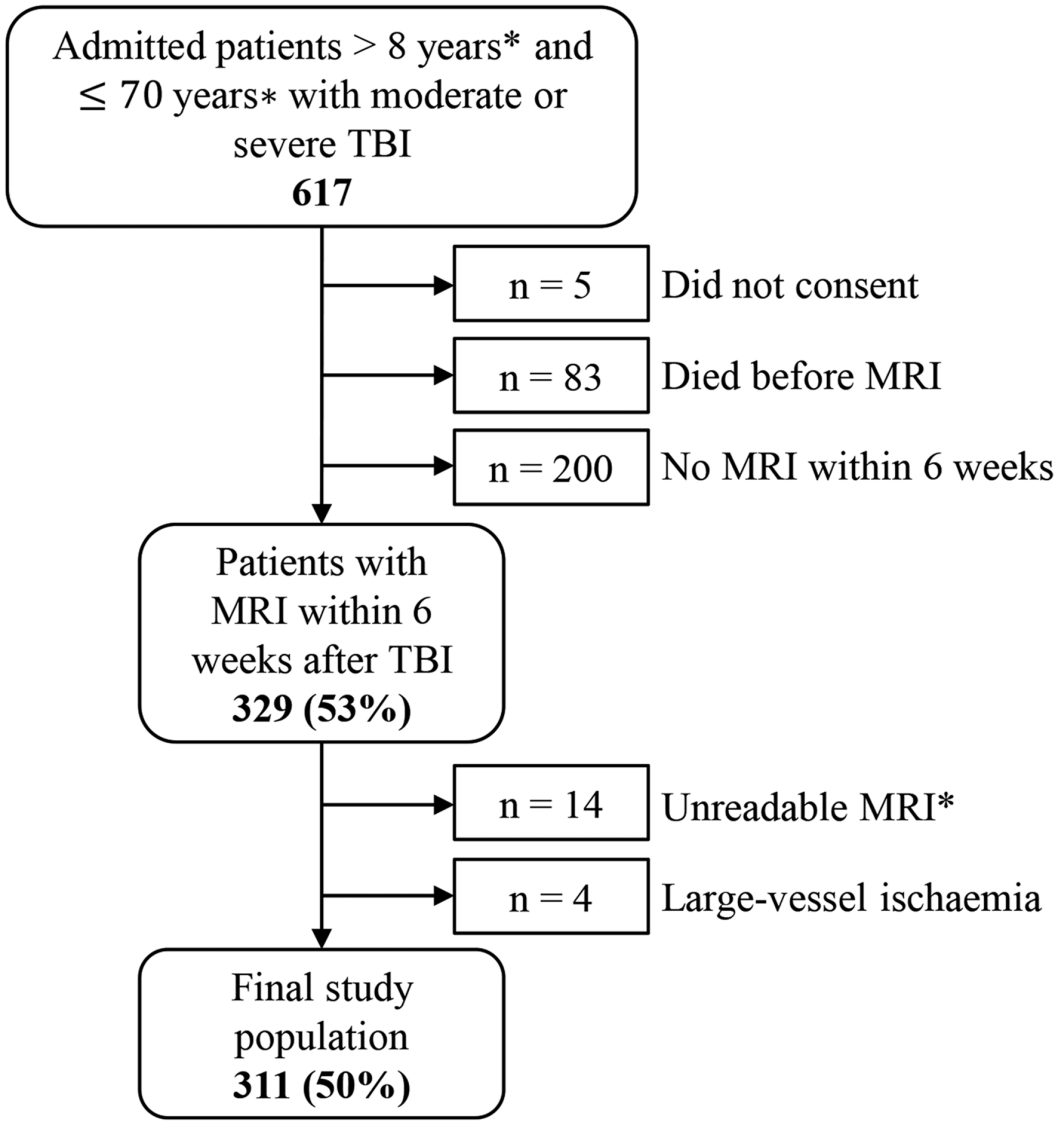
Flowchart showing inclusion and exclusion for this study. * For more details, we refer to the material and methods section in the manuscript. TBI = traumatic brain injury, MRI = magnetic resonance imaging

Patients admitted to St. Olavs Hospital, Trondheim University Hospital (Norway), between 01.10.2004 and 01.10.2019 (15 years) with moderate or severe TBI were included in this study (Fig. 1). Trondheim University Hospital is the only regional Level 1 trauma center in Central Norway covering a population of 748 226 inhabitants (01.01.2023). The patients were classified as having moderate or severe TBI according to the head injury severity scale (HISS) classification, which is based on the GCS score [2]. Moderate TBI was defined as a patient with a GCS score ranging from 9 to 13 or a patient with a GCS score of 14 or 15 and with loss of consciousness > 5 minutes (*n* = 6). Severe TBI patients had a GCS score ≤ 8.

### Inclusion and exclusion criteria

The inclusion of patients with moderate to severe TBI is illustrated in Fig. 1. The exclusion criteria were as follows: 1) patients < 8 years, due to challenges in assessing GCS and the need for general anaesthesia for MRI; 2) patients ≥70 years to reduce the influence of age-related nonspecific white matter hyperintensities on MRI [17]; 3) patients with MRI performed later than six weeks post-injury, due to known attenuation of TAI lesions and reduced detectability of lesions on MRI over time [18]; 4) patients with more than one missing or unreadable MRI sequence due to artifacts of the three MRI sequences considered essential for evaluation of TAI (see later); and 5) patients with acute ischemia/infarction in large vessel territories of the brain detected on diffusion weighted imaging (DWI), due to uncertainty around the cause and effect of TBI.

### Injury-related variables

Mechanisms of injury were classified into road traffic accidents, falls, struck by an object or violence and other/unknown. Prehospital or admission GCS scores as well as intubation statuses were recorded. Pupil dilation at admission was registered as normal, unilaterally or bilaterally dilated or not testable. Secondary events (hypoxia [O_2_ saturation below 92%] and hypotension [systolic blood pressure < 90 mm Hg] before or after admission to the hospital) were also recorded. Admission CT scans were reviewed by senior consultants in radiology (I.H.S., S.D., K.G.M., A.M.F.H.) and classified according to the Marshall CT score [19]. Intracranial mass lesion > 25 ml was defined as a Marshall CT score of 5 or 6.

### MRI scanning protocol

Patients were scanned at 3T (*n* = 38), 1.5T (*n* = 266) or 1T (*n* = 7) using a clinical protocol agreed upon before the start of the study. Fluid-attenuated inversion recovery (FLAIR), diffusion–weighted imaging (DWI), and either T2*-weighted gradient echo (GRE) or susceptibility-weighted imaging (SWI) were among the sequences included in the MRI protocol. For more details on the MRI scanning protocol, we refer to earlier studies [20, 21].

### Patients with TAI based on early MRI readings

**Fig. 2:**
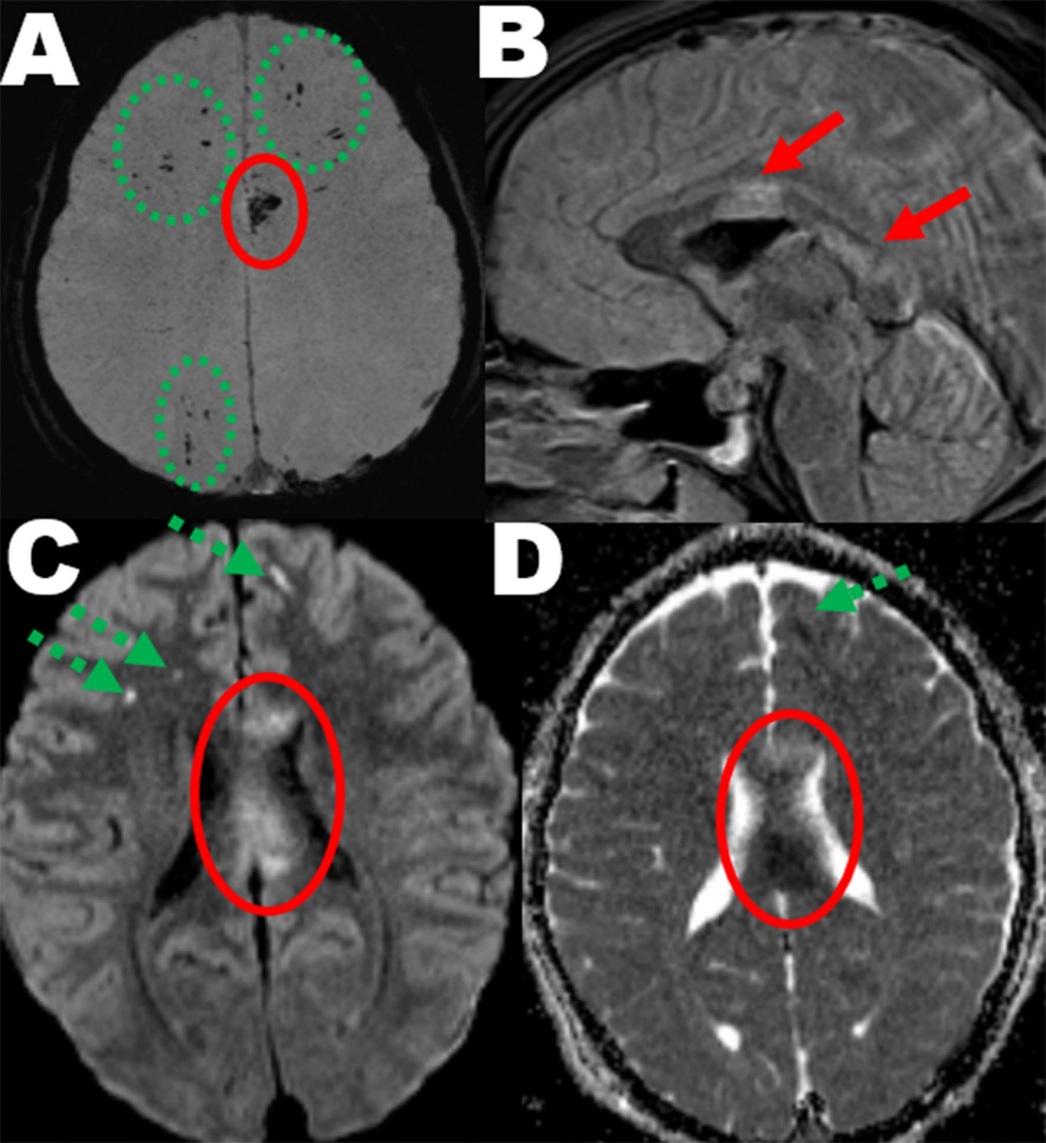
Findings of traumatic axonal injury in three different MRI sequences. A young male was admitted with GCS score 12 to the university hospital after a road traffic accident. CT in the acute phase was normal. 1.5T MRI was performed two days post-injury and showed traumatic axonal injury (TAI): **A**) Axial susceptibility weighted imaging (SWI) maximum intensity projection showing multiple traumatic microbleeds as sign of micro-haemorrhagic TAI in the hemispheric white matter of both frontal lobes and right parietal lobe (dashed green rings) and corpus callosum (continuous red ring). **B**) Sagittal FLAIR image showing TAI lesions in truncus and splenium of corpus callosum (continuous red arrows). **C**) The b1000 image of the diffusion weighted imaging (DWI) also showed hyperintense TAI lesions in the white matter of both frontal lobes (dashed green arrows, also corresponding to the traumatic microbleeds in image A) and corpus callosum (red continuous ring). **D**) The apparent diffusion coefficient (ADC) image at the same level as the b1000 image (image C) shows low diffusion of both one of the hemispheric TAI lesions (dashed green arrow) and most of the corpus callosum lesion (continuous red arrow)

Three MRI sequences were considered essential for assessing and classifying TAI: FLAIR, DWI, and either T2*GRE or SWI. On FLAIR and DWI, non-haemorrhagic TAI lesions can be detected, whereas on T2*GRE or SWI, we can detect micro-haemorrhagic TAI lesions [22, 23]. TAI was assessed across all these three sequences, and both micro-haemorrhagic and non-haemorrhagic lesions were included in the evaluation. We have shown the early MRI findings from one of the patients in this study in Fig. 2, to illustrate how signs of TAI can look like in different early MRI sequences. MRI scans were read and annotated in a blinded manner for clinical information by senior consultants in radiology (K.G.M., A.M.H.F., E.H.S., Ø.O. and S.A.B.). The MRI reports were considered “the gold standard” in this study. The TAI grade (0–3) was registered for all patients according to the standard TAI grading [10]. More detailed information on the MRI readings is presented in previously published papers [20, 21].

### Abbreviated injury scale (AIS) scoring and AIS head score

At the study hospital, the AIS1998 was the version used in the first year of the study period (*n* = 32), and the AIS2005/2008 version was used during the remaining study period (*n* = 279) [24, 25]. The latest update of the AIS is the edition from 2015, but in this version, the TAI scoring changed (see below paragraph). The AIS2005/2008 version is still used today at all trauma centres in Norway. It is well known from the literature that the AIS version used varies across institutions [26, 27].

Patients were AIS coded after admission by S.H., K.G.M., or I.A.B. under supervision by K.G.M. Coders in our study conferred with hospital trauma coordinators certified in AIS scoring to avoid coding discrepancies. The main AIS severity scores used for this study were for the body region “head”, hereafter called the AIS head score.

### Coding of traumatic axonal injury into the AIS head score: AIS-TAI coding

The AIS Dictionary uses the term DAI, but throughout this paper, we have used the interchangeable term TAI, which is increasingly used in both research and clinical practice. The developments within TAI coding in the AIS dictionaries from the 1990 to 2015 editions are presented in Table 1. In the AIS2005/2008 editions, TAI is defined as “a clinicopathological complex with immediate and prolonged coma due to widespread damage to axons and other neuronal processes in the brain”, and there are two separate code sets with distinct ways of classifying TAI [24, 25]: Code set 1 represents a modification of the standard TAI grading by Adams based on anatomical location [9], whereas code set 2 represents a severity grading based on the length of unconsciousness succeeding the injury (Table 1). In both code sets, all the TAI codes have high severity scores, either score 4 or 5. Nevertheless, to be able to code TAI with these code sets, three criteria must be met beforehand according to the AIS dictionaries: 1) the *coma* criterion (coma preceding more than six hours following immediately after injury), 2) the *no sedation* criterion and 3) the *no mass lesion* criterion (no intracranial mass lesion).

**Table 1:** Overview of TAI codes in the different versions of the AIS Dictionary from 1990 - 2015. All TAI codes independent on version have either severity score 4 or 5

	AIS 1990 and 1998	AIS 2005/2008	AIS 2015^d^
**AIS code set 1**	**DAI** ^a^ **classified based on anatomical location on imaging or by pathological examination**		
	14XXXX.5 DAI Cerebellum		
	14XXXX.5 DAI Cerebrum	14XXXX.4 DAI NFS	
		14XXXX.4 DAI confined to white matter^b^ or basal ganglia	
		14XXXX.5 DAI involving corpus callosum	
**AIS code set 2**	**DAI classified based on length of LOC / coma** ^c^ **and any brainstem signs**		
		16XXXX.4 DAI NFS	
		16XXXX.4 DAI with LOC / Coma 6-24 h (mild DAI)	
		16XXXX.5 DAI with LOC / Coma >24 h NFS	
		16XXXX.5 DAI with LOC / Coma >24 h without brainstem signs (moderate DAI)	
		16XXXX.5 DAI with LOC / Coma >24 h with brainstem signs (severe DAI)	

AIS = abbreviated injury scale TAI = traumatic axonal injury, DAI = diffuse axonal injury, NFS = not further specified, LOC = loss of consciousness, brainstem signs = decerebrate or decorticate signs (clinical characteristics are not further specified by AIS). Complete codes can be found in the respected AIS dictionaries, which can be purchased on the American Association of Automotive Medicine’s (AAAM’s) website

^a^AIS use the term DAI (in replacement of TAI), but the terms are used interchangeably in literature

^b^White matter refers to the white matter of the cerebral or cerebellar hemispheres

^c^Coma and LOC (loss of consciousness) have been accredited slightly different definitions in AIS and has been used interchangeably in different versions of the AIS Dictionary. In the AIS 2005 and 2008 update version LOC is used and in the AIS 2015 coma is used, for simplicity we have combined the terms

^d^In the AIS 2015 version only AIS codes based on coma and brainstem signs (AIS code set 2) can be used

### Defining patients eligible for the original AIS-TAI coding

The first author (IAB) used the abovementioned three criteria (i.e., coma, no sedation and no mass lesion) to assess whether patients were eligible for a TAI code using the *original AIS-TAI criteria.* The patients were categorized in the following manner: 1) patients with the lowest GCS score ≥ 9 prehospital or at admission (equals moderate TBI in the present study) were excluded because of violation of the *coma* criterion. 2) sedated and/or intubated patients were excluded because of violation of the *no sedation* criterion, since all intubated patients were sedated. In our health system, TBI patients in need of intubation are undergoing rapid sequence induction with the use of sedation performed by anesthesiologists both in pre- and in-hospital settings. 3) Patients with Marshall CT scores of 5 or 6 on admission CT images (mass lesion > 25 ml) were excluded because of violation of the *no mass lesion* criterion. If considered eligible, the AIS head scores were obtained and assessed with respect to the AIS codes in the AIS2005/2008 versions (Table 1).

#### A pragmatic AIS-TAI coding

A *pragmatic AIS-TAI coding* approach is used at the study hospital, since many patients are not eligible for a TAI code based on the *original AIS-TAI coding* despite having signs of TAI on MRI or even on CT. This consensus-based approach has been used by AIS coders at the Trondheim University Hospital since the start of the study. In this approach, moderate TBI (GCS score ≥ 9), sedated and/or intubated TBI patients and TBI patients with concurrent mass lesions could also be assigned a TAI code if deemed appropriate. The AIS coder further decided if TAI should be included as a separate code as part of the AIS head score or not. In this approach, we used the AIS2005/2008 version, and the AIS coder could not use the TAI codes based on length of coma or brainstem signs (code set 2, Table 1). We only used TAI codes based on anatomical location on early MRI (white matter, basal ganglia or corpus callosum) to simplify the AIS coding (code set 1, Table 1). This code set is not available in the AIS2015 version, which means that TAI coding is only based on coma/loss of consciousness and brainstem signs in this version. Since the AIS coding was performed months after the injury, the early MRI and the MRI reports were already available for the AIS coder during scoring.

### Injury severity scoring (ISS) and new injury severity scoring (NISS)

For the assessment of overall injury severity in patients with multiple traumatic injuries, the injury severity score (ISS) and new injury severity score (NISS) were also used [28, 29]. The ISS is the sum of the squares of the three highest AIS severity scores from six different body regions, whereas the NISS is a similar score where the sum of the squares of the three highest AIS severity scores is added regardless of body region. The ISS and NISS both range from zero (no injury) to 75 (worst outcome).

### Interrater analyses for injury severity scores and MRI readings

Interrater analyses were performed, comparing AIS head scores as well as ISS and NISS, for the three different AIS coders. Twenty different patients were randomly selected and rescored by each of the AIS coders (S.H., K.G.M., and I.A.B.), who were blinded to the information on scores provided by the other AIS coders. As a quality assurance, we also performed an interrater analysis of 16 randomly selected patients, comparing the scores obtained by I.A.B. with the scores obtained by the hospital trauma coordinator certified in AIS scoring at Trondheim University Hospital at the time (M.D.).

### Statistical analyses

Statistical analyses were performed using SPSS (IBM Corporation, released 2020. SPSS Statistics for Windows, Version 29, IBM Corporation, Armonk, NY, USA). Categorical variables are presented as frequencies with percentages. If not normally distributed, the data are presented as medians and interquartile ranges. If the data were normally distributed, the data are presented with means and standard deviations. For *the pragmatic AIS-TAI coding* approach, we estimated the sensitivity, specificity, positive predictive value (PPV) and negative predictive value (NPV), with findings on early MRI as the gold standard.

In the interrater analyses, an intraclass correlation coefficient (ICC) was calculated where a two-way mixed model was applied using absolute agreement. An ICC < 0.40 indicated poor agreement, 0.40–0.59 fair, 0.60–0.74 good and 0.75–1.0 excellent. Interrater analyses for MRI readings, including the presence of TAI and the TAI grade, are reported in a previously published paper [21]. 95% confidence intervals (CIs) were calculated for selected variables. The significance level was set to *p* < 0.05.

## Results

### Participant characteristics

The characteristics of the 311 included patients are presented in Table 2. The median age was 30 years, and the male proportion was 79%. The median AIS head score for the overall population was 4. The median number of days from injury to MRI was 9 for the overall population: 6 days for moderate TBI patients and 12 days for severe TBI patients.

### TAI on early MRI

A total of 249 patients (80%) had TAI on early MRI, where 24% had standard TAI grade 2 and 27% had standard TAI grade 3 with lesions in the brainstem (Table 2).

**Table 2:** Patient characteristics

	Total TBI MRI cohort	Moderate TBI (GCS 9 – 13)^a^	Severe TBI (GCS 3 – 8)
	n=311 (100)	n=156 (50)	n=155 (50)
**Age **(in years), median (IQR)	30 (21-48)	35 (22-53)	27 (20-43)
[min, max, range]	[9, 70, 61]	[9, 70, 61]	[9, 69, 60]
Gender			
Male	244 (79)	117 (75)	127 (82)
Female	67 (22)	39 (25)	28 (18)
**Cause of injury**			
Road traffic accident	160 (51)	70 (45)	90 (58)
Fall	111 (36)	59 (38)	52 (34)
Struck by object or violence	16 (5)	7 (5)	9 (6)
Other or unknown	24 (8)	20 (13)	4 (3)
**GCS score**, median (IQR) [missing]	9 (6-12) [2]	12 (10-13) [1]	6 (4-7) [1]
[min, max, range]	[3, 15, 12]	[9, 15^a^, 6]	[3, 8, 5]
**Pupil dilation at admission**	41 (13)	5 (3)	36 (23)
Unilateral	33 (11)	4 (3)	29 (19)
Bilateral	8 (3)	1 (1)	7 (5)
Not testable	4 (1)	3 (2)	1 (1)
**Intubation** ^b^	210 (68)	60 (39)	150 (97)
**AIS head score**, median (IQR)	4 (3-5)	4 (3-5)	5 (4-5)
**ISS score**, median (IQR)	25 (17-32)	20 (13-26)	29 (25-35)
**NISS score**, median (IQR)	34 (26-43)	27 (20-38)	41 (33-50)
**Secondary events** ^c^	76 (24)	17 (11)	59 (38)
**Marshall CT score**, median (IQR)	2 (2-3)	2 (2-3)	2 (2-5)
Score 1	42 (14)	24 (15)	18 (12)
Score 2	168 (54)	92 (59)	76 (49)
Score 3	28 (9)	10 (6)	18 (12)
Score 4	4 (1)	2 (1)	2 (1)
Score 5	60 (19)	23 (15)	37 (24)
Score 6	9 (3)	5 (3)	4 (3)
**Intracranial mass lesion >25 ml** ^d^	69 (22)	28 (18)	41 (27)
**Days from injury to MRI**, median (IQR)	9 (4-20)	6 (3-16)	12 (7-21)
**Patients with TAI on MRI**	249 (80)	110 (71)	139 (90)
No TAI	62 (20)	46 (29)	16 (10)
TAI grade 1	88 (28)	57 (37)	31 (20)
TAI grade 2	76 (24)	31 (20)	45 (29)
TAI grade 3	85 (27)	22 (14)	63 (41)

Values are presented as numbers (%) unless otherwise specified. TBI = traumatic brain injury, MRI = magnetic resonance imaging, GCS = Glasgow coma scale, AIS = abbreviated injury scale, ISS = injury severity score, NISS = new injury severity score, TAI = traumatic axonal injury, HISS = head injury severity scale

^a^n = 6 patients with GCS 14–15 included as moderate TBI according to HISS classification

^b^Prehospital or at admission to hospital

^c^Defined as hypoxia or hypotension before or at admission to hospital

^d^Defined as Marshall CT score 5–6

### TAI coding with the original AIS-TAI criteria

**Fig. 3:**
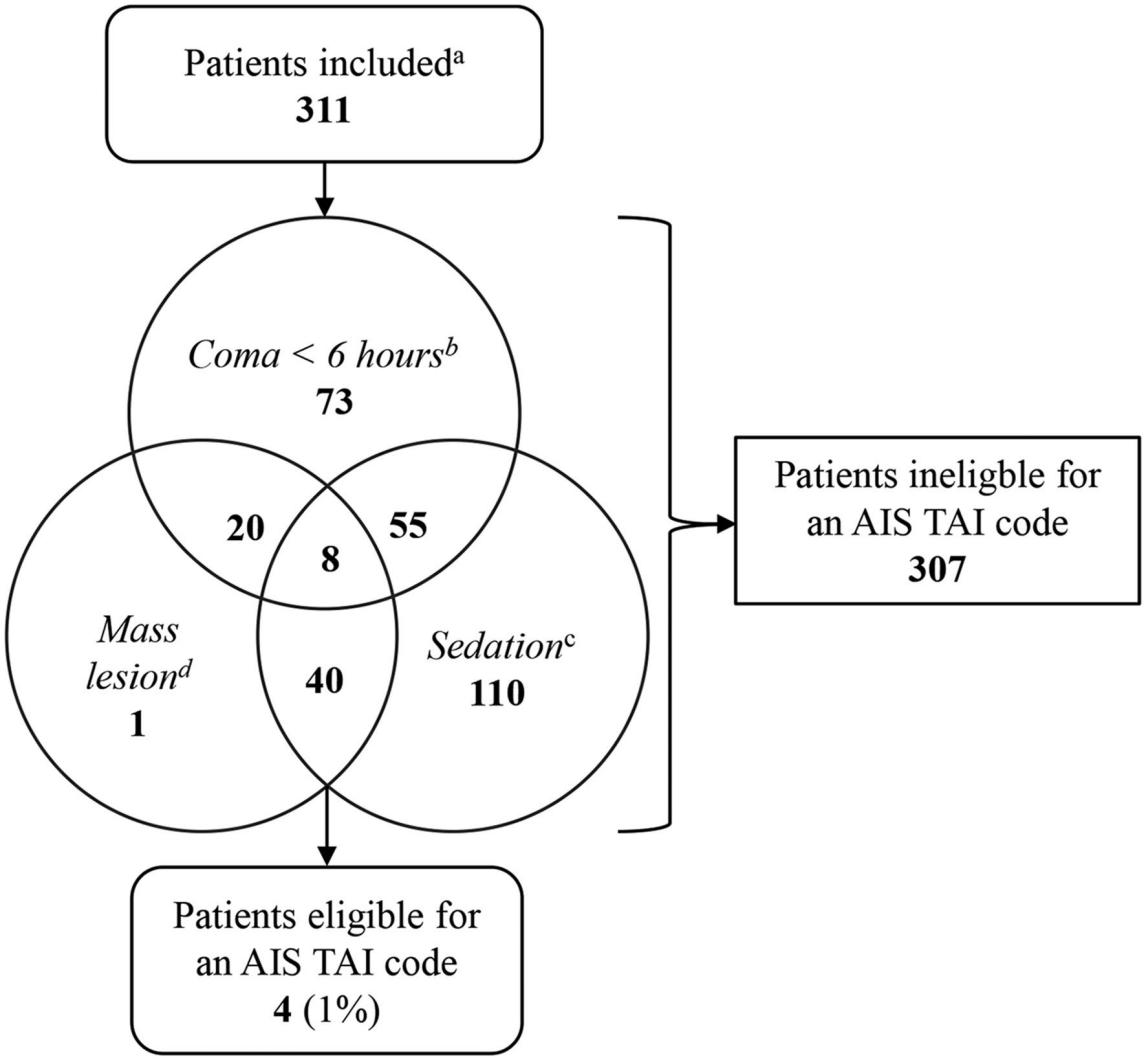
Venn diagram shows patient eligibility for coding of TAI according to *the original AIS-TAI criteria*. The number of patients violating the original AIS coding criteria are shown distributed on each criterion and combinations of criteria, respectively. Violation of criteria are defined in the “Materials and method” section and related to 1) *coma*, 2) *no sedation* and 3) *no mass lesion.*^a^Overall inclusion criteria in the study population are illustrated in Fig. 1 and described in detail in the “Materials and method” section ^b^Coma is defined as lowest GCS score ≤ 8 prehospital and at admission to university hospital. This group therefore corresponds to the moderate TBI group. ^c^Sedation comprises patients who are intubated or sedated at admission to university hospital (*n* = 3 patients were sedated but not intubated, otherwise this group correspond to the intubated group in our cohort) ^d^Mass lesion is defined as Marshall CT score 5 or 6 on admission CT. AIS = abbreviated injury scale, AIS TAI code = code for traumatic axonal injury, also called diffuse axonal injury in the AIS Dictionary

Only four of the 249 patients (1%) were found eligible to be assigned a TAI code using the *original AIS-TAI criteria* (Fig. 3). All four patients had TAI grade 1 on early MRI.

### TAI coding with the pragmatic AIS-TAI coding approach

A total of 125 patients (40%) were assigned a TAI code using the *pragmatic AIS-TAI coding* approach (Table 3). Since 249 patients had TAI on MRI, this approach showed a detection sensitivity of 50% and a specificity of 100%. The negative predictive value was 33%, and the positive predictive value was 100%. Among the 186 patients with no AIS-TAI code, 124 patients (67%) had TAI on MRI, whereas 63 (34%) had TAI grade 2 or TAI grade 3 (Table 3).

**Table 3:** Distribution of TAI codes in the pragmatic AIS-TAI coding approach, in relation to TAI lesions detected on early head MRI, also including subgroups with different standard TAI grades and TAI codes with severity score 4 or 5. Data presented with numbers (%). TAI on early MRI was considered the gold standard in this study, the AIS scorers could in the pragmatic AIS-TAI coding also include early MRI findings of TAI if the scorer found it appropriate to include them in relation to the available codes in the AIS Dictionary

	Detection of TAI on early head MRI (gold standard)					
Coding of TAI in the pragmatic AIS-TAI coding approach	No TAI	TAI	TAI grade 1	TAI grade 2	TAI grade 3	Total numbers
No TAI code	62 (33)	124 (67)	61 (33)	33 (18)	30 (16)	186 (60)
TAI code^a^	0 (0)	125 (100)	27 (22)	43 (34)	55 (44)	125 (40)
TAI code with severity score 4	0 (0)	32 (100)	20 (63)	6 (19)	6 (19)	32 (10)
TAI code with severity score 5	0 (0)	93 (100)	7 (8)	37 (40)	49 (53)	93 (30)
Total Numbers	62 (20)	249 (80)	88 (28)	76 (24)	85 (27)	311 (100)

TAI = traumatic axonal injury, MRI = magnetic resonance imaging, AIS = abbreviated injury scale

^a^All TAI codes in the AIS Dictionary are either severity score 4 or 5

### Interrater analyses

The interrater analyses revealed excellent agreement between the different AIS coders for the AIS head score, ISS and NISS, with ICCs ranging from 0.83 to 0.93 (Table 4). Interrater analyses for MRI readings of TAI were reported in a previous paper where the positive and negative agreement for the presence of TAI was 0.90 (95% CI 0.77–0.95) and for the absence of TAI was 0.69 (95% CI 0.42–0.84) [21]. The ICC for the classification of the standard TAI grade on early MRI was excellent (0.78).

**Table 4:** Intraclass correlation coefficients (ICC) for different AIS coders with respect to AIS head score, ISS and NISS

Coders	AIS head	C.I.^a^	ISS	C.I.^a^	NISS	C.I.^a^
SH, IAB^b^	0.834	0.586–0.934	0.923	0.808–0.969	0.877	0.692–0.951
KGM, IAB^b^	0.930	0.823–0.972	0.922	0.799–0.969	0.897	0.742–0.959
MD, IAB^c^	0.934	0.782–0.978	0.822	0.481–0.938	0.950	0.815–0.984

AIS = abbreviated injury scale, ISS = injury severity score, NISS = new injury severity score. SH, IAB, KGM and MD are abbreviations for the AIS coders conducting the AIS severity scores, ISS and NISS

^a^95% confidence intervals for the respective intraclass correlation coefficients

^b^20 subjects were randomly chosen for each rater

^c^16 subjects were randomly chosen for each rater

## Discussion

In this prospective study on moderate and severe TBI, we found that only 1% (four patients) would be eligible for a TAI code according to *the original AIS-TAI criteria*. Among all included patients, 80% had signs of TAI on their early MRI. When using a *pragmatic AIS-TAI coding* approach, still only half of the patients with signs of TAI on early MRI were classified with a TAI code as part of their AIS head score. Our study shows that the current TAI coding practices in the AIS exclude the majority of patients from having a TAI code, which does not reflect the findings of TAI on early MRI. We suggest a revision of the AIS Dictionary, where TAI coding is better defined and with instructions on how to include early MRI findings since MRI is increasingly performed in the early phase after moderate and severe TBI.

### Eligibility to obtain a TAI code using the original AIS-TAI criteria

Only four patients were eligible for a TAI code according to the *original AIS-TAI criteria*, which means that almost all patients who demonstrated TAI on early MRI were excluded from receiving a TAI code as part of their AIS head score. All four patients who were eligible for a TAI code according to the AIS Dictionary had only TAI grade 1 on early MRI. Thus importantly, being eligible to receive a TAI code did not reflect the severity of the TAI injury. This result is mostly explained by the exclusion of sedated and intubated patients. Hence, the most injured TBI patients, who are known to have higher TAI grades [20, 30], cannot be considered for a TAI code in *the original AIS-TAI criteria*.

This is the first study to quantify findings in detail and thereby demonstrate the shortcomings of the current assignment of TAI codes as part of the AIS head score. This is in accordance with the study by Bågenholm et al., who recently raised concerns regarding discrepancies in how TAI is diagnosed radiologically and how it is coded according to the AIS2005/2008 version [15]. We argue that the criteria in the AIS Dictionary related to coma preceding more than six hours and no sedation or concurrent mass lesions make AIS coders incapable of assigning patients with TAI codes as part of the AIS head score.

#### A pragmatic AIS-TAI coding approach

Compared with *the original AIS-TAI coding approach, a pragmatic AIS-TAI coding* approach markedly improved the identification of patients with TAI, as half of the patients with TAI on early MRI were given a TAI code as part of the AIS head score. We assume that similar pragmatic AIS-TAI coding approaches exist elsewhere world-wide, making AIS coders able to code TAI in practice. Nevertheless, half of the patients were not given a TAI code even if early MRI showed signs of TAI. The reason why AIS coders still did not include a TAI code as part of the AIS head score might be because they think that 1) the AIS head severity score associated with each AIS-TAI code does not reflect the presumed severity of the TAI and/or 2) the available AIS-TAI codes do not reflect the location of the TAI on clinical MRI. Only TAI codes with high injury severity scores exist (scores of either 4 or 5), and the anatomical locations do not comply with the TAI gradings used today [9, 10] or the recently proposed gradings of TAI on MRI [20, 31]. Additionally, the focus of the AIS Dictionary is much stronger in terms of clinical status than imaging findings, which probably makes AIS coders uncertain about including MRI findings of TAI as part of the AIS head score. This is further emphasized in the latest AIS2015 revision, where only code sets (code set 2, Table 1) based on clinical status can be used. TAI codes based on anatomical locations (code set 1, Table 1) were removed from the AIS2015 version. Interestingly, the AIS2015 version has still after ten years, not been adopted for use at trauma centers in Norway. Different strategies on how to code TAI might also hamper the ability to compare the prevalence of TAI in different health systems and thereby affect therapeutic approaches and follow-up of these patients.

We found high agreement between the AIS head score, ISS and NISS among AIS coders at our institution, using the *pragmatic AIS-TAI coding* approach. This is consistent with earlier studies, where the largest difference between AIS coders is found between institutions [15, 16].

### Future TAI coding practice in the AIS head score

Based on our findings, adhering to the *original AIS-TAI criteria*, will lead to a very large underestimation of the prevalence of TAI, which is an important type of injury in TBI. We therefore suggest a TAI coding as part of the AIS head score that better reflects the findings of TAI on head MRI. We argue that the AIS Dictionary’s criteria to code TAI do not reflect how TAI is internationally diagnosed today: as an imaging finding reported and graded on early MRI [8, 20]. We also know from recent MRI studies that both the presence and grade of TAI is important for the patient outcome [20, 31], supporting adding more specific MRI-TAI codes to the AIS Dictionary. There is a lack of differentiation of severity scores to reflect the different TAI grades today, where only AIS severity scores of 4 and 5 are available. Patients with a TAI grade 1 have more favourable outcomes than patients with a TAI grade of 2 or 3 [12, 30, 32]. Therefore, we argue that the severity scores available in the AIS Dictionary should reflect the different grades used to report TAI on MRI. This could be according to the today commonly used standard TAI grading [9, 10], where patients with TAI grades of 1, 2 and 3 could be assigned severity scores of 3, 4 and 5, respectively. However, we recently published the Trondheim TAI-MRI grading, which better reflects injury severity and patient outcome [20]. Compared with standard TAI grading, this newly proposed grading system takes into consideration the bilaterality of TAI lesions and the location of TAI lesions in the brainstem and includes TAI lesions in the thalami and basal ganglia, all of which are known to be important MRI biomarkers for overall prognosis [20]. We argue that future and improved TAI grading systems should continuously be considered for incorporation in updated versions of the AIS Dictionary. The Trondheim TAI-MRI grade consists of 5 grades, since AIS codes also have 5 severity scores, there could be a natural recoding if this coding system should be included. However, performing head MRI in the early weeks is not accessible in all countries or trauma centers, due to limited MRI availability or resources. In such cases, alternative ways of coding coma, brainstem signs and/or suspicion of TAI as part of the AIS head score must still be an option in an updated AIS Dictionary.

### Strengths and limitations of the study

The strengths of this study are the prospective study design and the large sample size where patients had a high rate of early head MRI within six weeks (most within 2 weeks). Because there were no new changes that would affect AIS head scoring from AIS2005 to AIS2008, the results of this study apply to both versions. Three different AIS coders were used in this study, where the interrater analyses all showed excellent agreement.

There are some limitations in this study. There are often selection biases in MRI cohorts; the most severely injured patients die before MRI, whereas less injured patients may be discharged or redistributed to other wards where there is less tradition of referring to MRI as part of the follow-up [33]. Thirty-two patients from the earliest part of our patient cohort were scored according to the AIS1998. Optimally all patients should have been scored according to the AIS2005/2008 version, but due to the small amount, we did not find it necessary to rescore these few patients retrospectively.

## Conclusion

In conclusion, we have demonstrated that by using the *original AIS-TAI criteria*, almost no patients will be eligible to be assigned with a TAI code as part of the AIS head score. By using a *pragmatic AIS-TAI coding* approach, the inclusion of TAI codes to the AIS head score was significantly better, but still only half of what was reported on early head MRI. We therefore suggest a revision of the AIS Dictionary to include an option for coding and grading of TAI based on findings from early MRI, rather than relying solely on clinical assessment of length of coma, which remains the only option in the last updated AIS2015 version. We also want to emphasize that future improved grading systems of TAI on early MRI should be continuously considered for incorporation in future AIS Dictionary revisions.

## Data Availability

Data collection was performed in a web-based data collection system administered by the faculty of Medicine and Health Sciences at the Norwegian University of Science and Technology (NTNU), Trondheim, Norway. According to national legislation, data cannot be shared openly. Data can be made available if formal collaboration is established and after appropriate approvals from national legislation.

## Abbreviations

TBI: Traumatic brain injury
MRI: Magnetic resonance imaging
CT: Computed tomography
TAI: Traumatic axonal injury
DAI: Diffuse axonal injury
AIS: Abbreviated injury scale
ISS: Injury severity score
NISS: New injury severity score
FLAIR: Fluid attenuated inversion recovery
DWI: Diffusion weighted imaging
GRE: Gradient echo
SWI: Susceptibility weighted imaging
CI: Confidence interval
IQR: Interquartile range
MIN: Minimum
MAX: Maximum
ML: Millilitres
GCS: Glasgow coma scale
HISS: Head injury severity scale
LOC: Loss of consciousness
NFS: Not further specified
AAAM: American Association of Automotive Medicine
